# Epigenetic modifications and transgenerational inheritance in women victims of violence (EWVV)

**DOI:** 10.1093/eep/dvaf025

**Published:** 2025-09-10

**Authors:** Donato Gemmati, Matteo Villanova, Fabio Scarpellini, Daniela Milani, Rossana Cecchi, Ajay Vikram Singh, Rosa Maria Gaudio, Veronica Tisato

**Affiliations:** Department of Translational Medicine, University of Ferrara, 44121 Ferrara, Italy; University Strategic Centre for Studies on Gender Medicine, University of Ferrara, 44121 Ferrara, Italy; Centre Haemostasis & Thrombosis, University of Ferrara, 44121 Ferrara, Italy; Department of Education Sciences, Roma Tre University, 00154 Rome, Italy; CERM-Hungaria, 00198 Rome, Italy; Department of Translational Medicine, University of Ferrara, 44121 Ferrara, Italy; Department of Biomedical, Metabolic and Neural Sciences, Institute of Legal Medicine, University of Modena, 41125 Modena, Italy; Department of Chemical and Product Safety, German Federal Institute for Risk Assessment (BfR), 10589 Berlin, Germany; Department of Translational Medicine, University of Ferrara, 44121 Ferrara, Italy; University Strategic Centre for Studies on Gender Medicine, University of Ferrara, 44121 Ferrara, Italy; Section of Legal Medicine and LTTA Centre, University of Ferrara, 44121 Ferrara, Italy; Department of Translational Medicine, University of Ferrara, 44121 Ferrara, Italy; University Strategic Centre for Studies on Gender Medicine, University of Ferrara, 44121 Ferrara, Italy; LTTA Centre, University of Ferrara, 44121 Ferrara, Italy

**Keywords:** epigenetics, methylome, PTSD, stress response, women violence, epidrugs, gender-based violence, coping, resilience

## Abstract

Female survivors of physical or psychological violence, including sexual violence, report significant long-term consequences defined as post-traumatic stress disorder (PTSD). Among these, depression, affective difficulties, anomalous behaviours, and worsened reproductive health may also affect offspring through transgenerational transmission involving primordial germ cells (PGCs) and/or through social transmission and acquisition of behavioural patterns from parent(s) to children. The concept of epigenomic modification involves several molecular targets that are sensitive to environmental stressors, which tune gene activity and expression. DNA methylation, histone acetylation, ncRNAs, telomere attrition, and mitochondrial dysfunction cooperate in maintaining homeostasis and may affect genes involved in key pathways, such as the hypothalamic–pituitary–adrenal axis, mediating the integrated homeostatic response to stressors. The most investigated genes were those implicated in neuroendocrine stress responses; dopamine, norepinephrine, and serotonin signalling; apoptosis; insulin secretion; neuroplasticity; reproduction; foetal growth; and cancer (e.g. *MAOA, BRSK2, ADCYAP1, BDNF, DRD2, IGF2, H19*). Additional investigated genes were those involved in other important functions, such as neuropeptide binding, immunoregulation, histone deacetylase/demethylase, inflammatory response, and serotonin uptake, yielding interesting but preliminary or not completely replicated findings (e.g. *CRHR1, FKBP5, KDM1A, NR3C1, PRTFDC1*, and *SLC6A4*). The assumption that epigenetic traits induced by negative experiences can be reversed by appropriate social, psychological, and pharmacological interventions has prompted the scientific community to investigate the relationship between epigenetic mechanisms and physical and psychological violence. This can help to identify direct links or epigenetic marks useful for optimizing personalized interventions encompassing the genetic, neuropsychiatric, social, and forensic medicolegal fields. Future research should be conducted with extreme caution to evaluate the long-term effects of such strategies and assess whether the immediate observed effects are maintained.

## Graphical Abstract

**Graphical Abstract:**
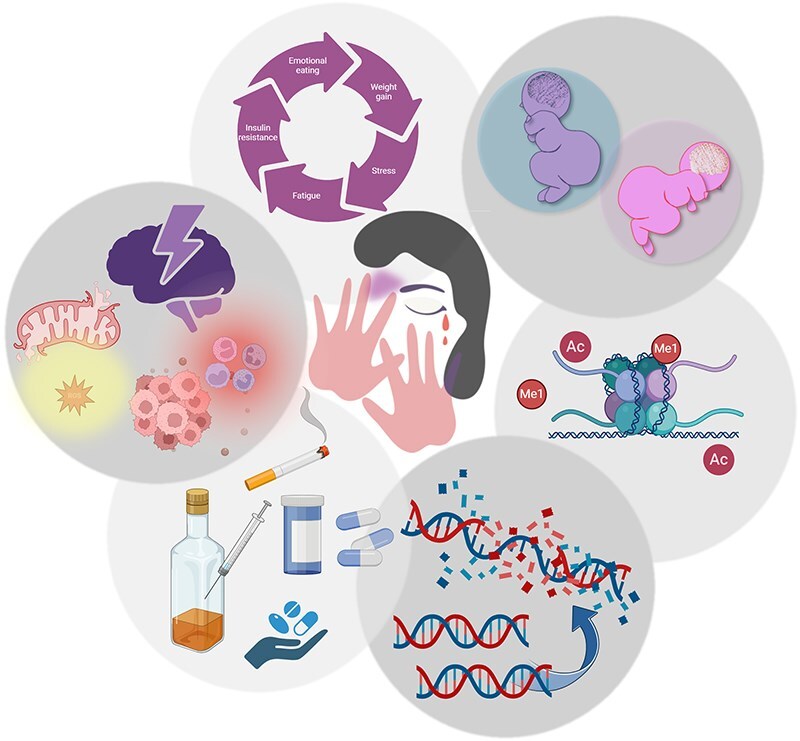

## Introduction

Epigenetics investigates genomic modifications that do not alter DNA sequences. Such environmentally acquired modifications may regulate the transcriptional landscape of cells in exposed individuals and in their offspring [1–4]. Thus, environmentally adapted epigenetic information may be transmitted through germline cells across generations. Although fascinating, extreme caution and attention should be paid to the interpretation of the underlying mechanisms before considering epigenetic inheritance or transmission in this context. Epigenetic modifications and observed phenotypes induced by environmental stressors via direct intrauterine exposure of the foetus are often incorrectly considered as transgenerational epigenetic inheritance. Intergenerational transmission should be instead considered, similar to that in exposed males or nonpregnant females. This is because somatic cells, gametes, primordial germ cells (PGCs), and developing embryos are directly exposed to stressors [5]. Unfortunately, the limited data available come from preclinical models and cannot be directly translated to humans, considering the different complexities and high rates of inbreeding [3, 6].

In recent years, increasing attention has been paid to the nongenetic transmission of traumatic experiences via epigenetic regulation across generations. Of the several circumstantial factors responsible for epigenetic modifications, exposure to trauma or stressful experiences, such as violence, has been considered a high priority.

The World Health Organization (WHO) states that approximately 30% of women are subjected to physical and/or sexual violence by an intimate partner (IPV) or a nonpartner. IPV includes physical, sexual, and emotional mistreatment [7] and is one of the most frequent forms of violence against women, touching couples across all socioeconomic and educational levels [8]. The United Nations Office on Drugs and Crime (UNODC) estimates that women killed constitute approximately 50% of all victims in their homes, and over 65% of all victims are IPV [9–11]. Accordingly, IPV is defined as ‘a behavior in any relationship that is used to gain or maintain power and control over an intimate partner’ [8].

Environmental and risk factors predisposing to violence against women embrace a wide range of conditions, including lower educational levels, previous violence during childhood or witnessing family violence, antisocial personality disorders, excessive male controlling behaviours towards females, lower income, use of alcohol, and several others [10, 12, 13]. Women who have experienced violence have increased rates of post-traumatic stress disorder (PTSD), including complex conditions such as depression, anxiety, mental health problems, and sleep or eating disorders [14–17]. Violence is also a cause of increased risk for physical conditions like cardiovascular diseases, hypertension, diabetes, fibromyalgia, asthma, and cancer, and reduced reproductive health and longevity [18–20]. Regarding addiction behaviour, female survivors usually consume more anxiolytics or antidepressants, smoke more cigarettes, and abuse alcohol or other types of drugs [11, 21, 22]. We describe a complex scenario to investigate; for example, it must be considered that all these harmful conditions may already exist before the violent episode in the victim or in her family, making it extremely difficult to distinguish and evaluate the exact genesis or timing of the PTSDs and/or the observed epigenetic modifications. Moreover, victims may react to experienced violence by engaging in or increasing harmful behaviours that can induce epigenetic modifications, change their diet, and change their habits, making it difficult to identify the causality between a specific violent event and the presence of that specific epigenetic mark.

Excessive chronic stress to which victims are exposed may be one of the most plausible pathways leading to negative health outcomes. Moreover, once exposure to acute mental and/or physical violence against women stops, the detrimental effects may persist, and women may continue to suffer mistreatment from their partners or perpetrators and face societal pressure [23, 24]. Although difficult to measure, the exact intensity and duration of violence are additional factors to consider for correct biological and epigenetic analyses [24].

The observation that such negative experiences and trauma may favour the establishment of pathological conditions and cause, via epigenetic mechanisms, transmissible (observable) marks in subsequent generations opens a previously unexplored branch of environmental (heritable) trait transmission [25–27]. Although speculative, there is evidence that long-lasting epigenetic marks may be reversed, unlike classical genetic traits, which are categorically invariable. It has been reported that unwanted epigenetic traits can be reversed by appropriate epigenome editing, also via social and psychological interventions, introducing the curable (epi)-genome concept [28, 29]. More effective and validated pharmacological interventions, namely epidrug treatments, are a class of molecules that target epigenetic marks by regulating epigenetic mechanisms, primarily through the activity of specific enzymes, such as DNMTs and HDACs, or by targeting mRNAs using specific miRNAs [30–32]. Epidrug-based approaches have demonstrated efficacy as anticancer agents [33–35], and dysregulation of epigenetic modifications can be involved in complex conditions like inflammation, obesity, type 2 diabetes, dyslipidaemia, cardiovascular diseases, neurological disorders, and metabolic disorders [36].

The overlap of these clinical conditions with PTSD suggests common mechanisms and etiopathogenesis; thus, epigenetic rebalancing may represent a potentially innovative strategy for the future treatment of women with PTSD. While the effectiveness of epidrugs in treating cancer is an existing reality, particularly when combined with standard chemotherapeutic agents, demonstrating promising effects in preclinical and clinical settings as cytostatic and cytotoxic compounds capable of reducing chemotherapy toxicity [37], epidrug-based treatment options for PTSD or neurological/psychiatric conditions are still in the early experimental stage. Nonetheless, epidrugs may have broad therapeutic potential for complex conditions with a strong environmental component. Their validation may take advantage of the development of disease-specific biomarkers associated with epigenetic changes in degenerative diseases [38–40], with the final aim of reversing detrimental epigenetic modifications via methylation rebalancing or including (but not limited to) miRNAs designed to restore normal gene expression dynamics [41–43].

**Figure 1.**
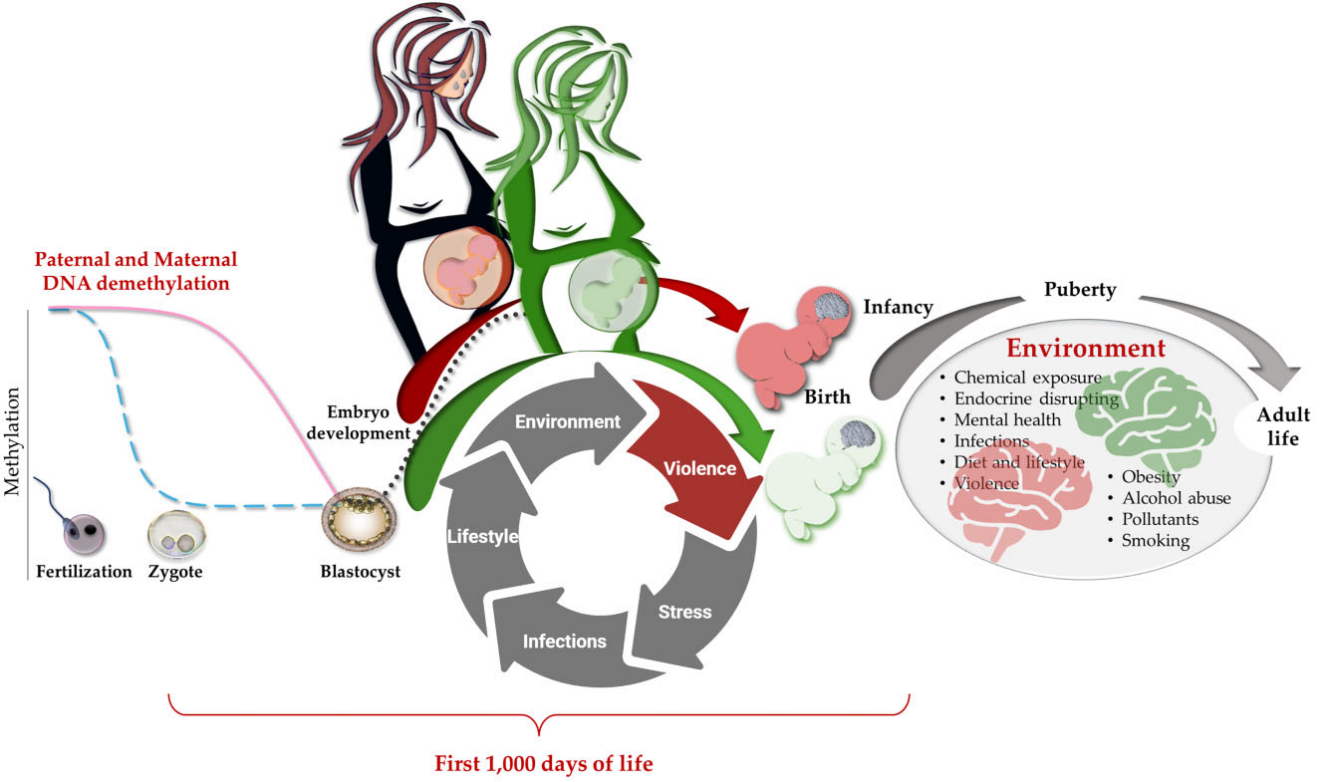
Landscape of normal and aberrant epigenetic modifications occurring from fertilization to adulthood: periconception, pregnancy, perinatal, and infancy (first 1000 days of life). On the left, DNA demethylation processes occurring during fertilization. Continuous pink line and blue dashed line indicate the female and male hemigenomes, respectively. *De novo* global genome methylation during the blastocyst phase is indicated by a dark dashed line. Modified from [94] created using BioRender.com.

Parental exposure to negative environmental actions, such as physical or psychological violence, natural disasters, pollution, infections, toxins, or cancer, can also interfere with the biological process of conception, from the epigenetic reprogramming of PGCs to the foetus development [44–46], up to childhood or adulthood, predisposing it to disease (Fig. 1). This should be properly considered for the correct categorization and interpretation of any generational transmission, as recently reported in Nature, discussing results from a study on the possible genetic inheritance of violence in Syrian refugees [5, 47].

Numerous interactions exist between epigenetic mechanisms (i.e. DNA methylation, histone acetylation, ncRNAs, and telomere attrition) and other biological pathways indirectly targeted by epidrugs. One of the most interesting aspects is the bidirectional interplay between epigenetics and mitochondria, as the most essential genes for mitochondrial functions are encoded by genomic DNA, entailing a synchronized interplay between the nucleus and mitochondria (i.e. mito-nuclear communication) [48].

Epigenetic changes or PTSD induced by major trauma, such as those observed in survivors of natural disasters, wars, holocausts, or terrorist attacks [49, 50], together with physical or psychological violence against children or adults, focus on common epigenetic mechanisms causing changes in gene activity or expression. This means that profound negative life experiences, particularly those in the early years of life [51–53], or other stressors such as cancer and associated treatments, can (semi)-permanently alter cellular biological processes with long-term effects on health and individual behaviour and can be passed on to offspring [54–56]. Although extensive correlation data support the concept that epigenetic mechanisms underlie biological embedding, causal data are still lacking in humans, and any attempt to link them is extremely challenging and complicated by the interference of additional exogenous or endogenous factors, such as age, sex, cell type, lifestyle, timing of experience, and DNA sequence [57]. Accordingly, a more appropriate and inclusive definition of epigenetics may be that reported by the NIH Epigenomics Roadmap Project initiative, which states that ‘Epigenetics refers to both heritable changes in gene activity and expression (in the progeny of cells or of individuals) and also stable, long-term alterations in the transcriptional potential of a cell that are not necessarily heritable’ [58, 59].

The main investigations in molecular processes refer to methyl group attachment or erasure in specific genome areas, which may cause hypermethylation or hypomethylation, respectively, according to mono-carbon unit availability [60]. These actions modify, promote, or repress transcriptional activity. Chromatin accessibility plays a major role and is strongly regulated by the repressive or active effects of histones, resulting in the closed or open status of specific parts of the genome, contributing to the tuning of gene expression. Finally, ncRNAs are a further level of mRNAs regulation, which, through specific interactions with the 3′UTR regions, determine the fate and efficiency of the final gene product by partially or completely blocking and degrading mature mRNAs. Therefore, we aimed to discuss the potential connections between biological epigenetic mechanisms and physical or psychological violence, with an ambitious attempt to find a direct link between stress-related disorders and epigenetic markers in women who have experienced physical, sexual, or psychological violence. Moreover, a set of genes and genome areas selected from those highlighted by available genome-wide association studies (GWAS) will also be discussed, hoping for the usefulness of candidate-specific molecular pathways and genes to elucidate these complex mechanisms [61–64]. Studying the epigenetics of violence against women may represent an advanced strategy to implement existing OMIC approaches aimed at optimizing personalized interventions and treatments that encompass the formal genomic, psychiatric, social, and medicolegal fields.

## Genes and epigenetic marks of violence

From a biological perspective, gamma-aminobutyric acid (GABA) and hypothalamic–pituitary–adrenal (HPA) axes play key roles in regulating environmental stress responses in mammals. GABA is a major inhibitory neurotransmitter that exerts regulatory effects on the HPA axis, resulting in a combined network that represents the major interface between stressors and the maintenance of homeostasis, avoiding stress-induced overactivation. Accordingly, more investigated genes are those encoding mediators involved in neuroendocrine stress responses, dopamine, norepinephrine, and serotonin signalling, with effects on insulin secretion, neuroplasticity, inflammation, and apoptosis, as recently reported [65, 66]. Specific comments on the primary roles and diseases associated with the investigated genes are provided below.

*MAOA* (Xp11.3), the monoamine oxidase-A gene, belongs to one of the two gene family members that encode mitochondrial enzymes that catalyze the oxidative deamination of amines such as dopamine, norepinephrine, and serotonin. This enzyme regulates normal synaptic function in the brain and exogenous and endogenous stress responses. *MAOA* mutations primarily cause Brunner syndrome, a disorder characterized by intellectual disability and aggressive behaviour associated with MAOA dysfunction. PTSD is characterized by other psychiatric and neurological disorders, including antisocial behaviour, which characterizes the clinical picture of PTSD [67–71]. *MAOA* gene methylation patterns in women who had experienced violence, such as sexual assault and/or physical attacks, during childhood, showed a slight increase in methylation levels in both exons and introns of the gene, whilst those who had been raped showed hypermethylation of the first exon [72, 73].

*BRSK2* (11p15.5) encodes brain-specific serine/threonine-protein kinase 2, which is crucial for neuronal polarization and axonogenesis. It has other functions like ATP/ATPase binding activity, magnesium ion binding activity, G2/M cycle transition of mitotic cells, apoptotic signalling in response to endoplasmic reticulum stress, and regulation of insulin secretion. This enzyme is highly expressed in the hippocampus and is associated with PTSD and memory formation. Modification of *BRSK2* methylation and expression may interfere with PTSD-related neurotransmitter levels [74, 75]. Anomalous methylation of this gene was recognized after a whole-genome analysis performed three months after a traumatic event by comparing women who were raped and developed or did not develop PTSD. Reduced *BRSK2* methylation levels in intron 4 have been found in PTSD and are directly related to symptom severity [76].

*ADCYAP1* (18p11.32) encodes pituitary adenylate cyclase-activating polypeptide (PACAP), which is further processed into multiple mature peptides actively involved in promoting adenylate cyclase (AC) and cyclic adenosine monophosphate (cAMP) levels, resulting in the transcriptional activation of target genes, including the key mediators of neuroendocrine stress responses. High PACAP levels are associated with severe PTSD symptoms by affecting the HPA axis and associated stress responses [77, 78]. In contrast with *BRSK2* in the same study [76], increased *ADCYAP1* methylation was observed within intron 1 of the gene associated with PTSD and symptom severity [76].

*BDNF* (11p14.1) encodes brain-derived neurotrophic factor, a member of the nerve growth factor family of proteins, which is a key regulator of synaptic transmission and neuroplasticity. It plays a role in stress response, learning, and memory maintenance [79]. The binding of this protein to its receptor promotes neuronal survival in the adult brain. Accordingly, this gene has reduced expression levels in Alzheimer’s, Parkinson’s, and Huntington’s diseases. Moreover, this gene plays a role in regulating the stress response and mood disorder biology, and increased methylation in the promoter has been observed among Vietnam War veterans with PTSD [80].

*DRD2* (11q23.2) encodes the D2 dopamine receptor subtype, a G-protein-coupled receptor that inhibits AC activity. Various mutations in this gene have been associated with schizophrenia, and missense mutations are responsible for myoclonic dystonia (MD). Alternative splicing produces transcript variants that encode different isoforms. Studies have shown that women with bulimia spectrum disorders (BSD) have hypermethylation of *the DRD2* promoter, in accordance with reduced dopamine levels. Moreover, women with BSD, characterized by a history of childhood sexual mistreatment, show higher *DRD2* methylation levels than those in the control group, suggesting persistent epigenetic modifications at this gene locus following sexual violence [81].

*IGF2* (11p15.5) encodes insulin-like growth factor 2, a member of the insulin family of growth factors involved in development and is a key regulator of foetal and placental growth. It is an imprinted gene expressed only in the paternal allele, and epigenetic changes at this locus are associated with Wilms’ tumour, Beckwith–Wiedemann syndrome, and Silver–Russell syndrome. *IGF2* also influences brain-associated conditions like memory, depression, and autism [82]. Alterations in *IGF2* expression have been observed in US military soldiers deployed to Afghanistan or Iraq who developed PTSD [83]. In a study involving female victims of sexual violence, the *IGF2* promoter showed higher methylation scores in women who developed PTSD symptoms [63].

*H19* (11p15.5) is located in an imprinted region of chromosome 11 close to *the IGF2* gene, and conversely to *IGF2*, it is expressed only on the maternally inherited chromosome, and its product is a long ncRNA that functions as a tumour suppressor. The proximity of the two genes allowed the regions to overlap and cover both the *IGF2* promoter and the binding region of the enhancer-blocking element upstream of the *H19* start site. As for the *IGF2* gene, mutations in this gene are associated with Beckwith–Wiedemann Syndrome and Wilms tumour, and these peculiar concurrences should recommend a combined investigation of the two genes. Although preliminary, studies on US soldiers have reported that those who did not develop PTSD showed reduced *H19* methylation after deployment [83, 84]. Adverse maternal childhood experiences and female discrimination predicted altered methylation of the *H19* and *IGF2* genes in offspring [85].

Besides the abovementioned genes, other candidate genes have also been investigated as targets for methylation fluctuations and/or PTSD establishment in the epigenome of individuals who experienced violence or their offspring. Among these, interesting additional results, although considered preliminary or not completely validated or replicated, account for the following selected genes: *CRHR1, FKBP5, KDM1A, NR3C1, PRTFDC1*, and *SLC6A4*, which are involved in other important functions, such as neuropeptide binding, the HPA axis, immunoregulation, histone deacetylase/demethylase, inflammatory response, cellular proliferation/differentiation, memory functions, and serotonin uptake [61, 62, 84]. Table 1 summarizes the data and links each gene to its main relevant characteristics.

**Table 1.** Main genes and locus involved in PTSD investigated in women after violence

Gene	Locus	Name	Product characteristics	Classical clinical phenotype
*MAOA*	Xp11.3	Monoamine oxidase A	Mitochondrial enzyme	Brunner syndrome, intellectual delay, ADHD, psychiatric disorders, ASD, antisocial behaviour
*BRSK2*	11p15.5	BR serine/threonine kinase 2	Protein kinase 2	Developmental and intellectual delay, autism, motor delay, feeding issues, seizures
*ADCYAP1*	18p11.32	Adenylate Cyclase Activating Polypeptide 1	Secretin/glucagon/vasoactive intestinal peptide family member	Regulate cellular stress response, abnormal stress response
*BDNF*	11p14.1	Brain Derived Neurotrophic Factor	Nerve growth factor family proteins	Reduced in AD, PD, and HD
*DRD2*	11q23.2	Dopamine receptor D2	G-protein coupled receptor	Possible association with schizophrenia and myoclonic dystonia
*IGF2*	11p15.5	Insulin like growth factor 2	Insulin family growth factors	Foetus growth restriction, Wilms tumour, Beckwith–Wiedemann syndrome, RMS, SRS
*H19*	11p15.5	H19 imprinted maternally expressed transcript	Long ncRNA tumour suppressor	Beckwith–Wiedemann syndrome and Wilms tumourigenesis
*CRHR1*	17q21.31	Corticotropin releasing hormone receptor 1	G-protein coupled receptor	Anomalous HPA
*FKBP5*	6p21.31	FKBP prolyl isomerase 5	Immunophilin protein family/glucocorticoid receptor co-chaperone	Depression, efficacy of antidepressant drugs, immuno-regulation
*KDM1A*	1p36.12	Lysine demethylase 1A	Nuclear protein component of HDM/HDAC complex	Cleft palate, psychomotor retardation, distinctive facial features
*NR3C1*	5q31.3	Nuclear receptor subfamily 3 group C member 1	Glucocorticoid receptor	Generalized glucocorticoid resistance
*PRTFDC1*	10p12.1	Phosphoribosyl transferase domain containing 1	Human HPRT homolog (inhibitor of hypoxanthine conversion)	Possible tumour suppressor gene, potential PTSD gene, childhood obesity
*SLC6A4*	17q11.2	Solute carrier family 6 member 4	Integral membrane protein (serotonin neurotransmitter recycle)	OCD, anxiety-related personality traits

ASD, autism spectrum disorders; ADHD, attention deficit hyperactivity disorders; RMS, rhabdomyosarcoma; AD, Alzheimer’s disease; PD, Parkinson’s disease; HD, Huntington’s disease; OCD, obsessive-compulsive disorder; SRS, Silver–Russell syndrome.

## Transgenerational epigenetic impact of violence and trauma

When adults are exposed to external stressors, the somatic cells and germlines of the foetus in pregnant females are directly exposed to possible inducible epigenetic modifications. Formally, they all belong to the F0 generation of exposed subjects, and their observable phenotypes (if any) can presume no inheritance. Furthermore, DNA methylation in mammals is globally reduced twice in each generation—immediately after fertilization and during PGC development. Therefore, it is difficult in mammals and virtually impossible in humans to exclude potential confounding elements, such as maternal contribution, components of seminal fluids, in utero changes, postnatal effects, lifestyle, or genetic predispositions, as the main cause of the observed epigenetic or phenotypic alterations [59].

Nonetheless, transgenerational epigenetic inheritance in humans is limited but possible, and the synergy between genetics and epigenetics may help identify specific trajectories of inheritance, particularly by complementing GWAS data with EWAS data to assess the relative contributions of the genome and epigenome to inheritance mechanisms and complex diseases [59]. Moreover, we cannot exclude the possibility that flaws in genes that remove (erase) or re-establish (rewrite) epigenetic marks are responsible for and/or cooperate in the establishment of epigenetic inheritance in mammals [86].

**Figure 2.**
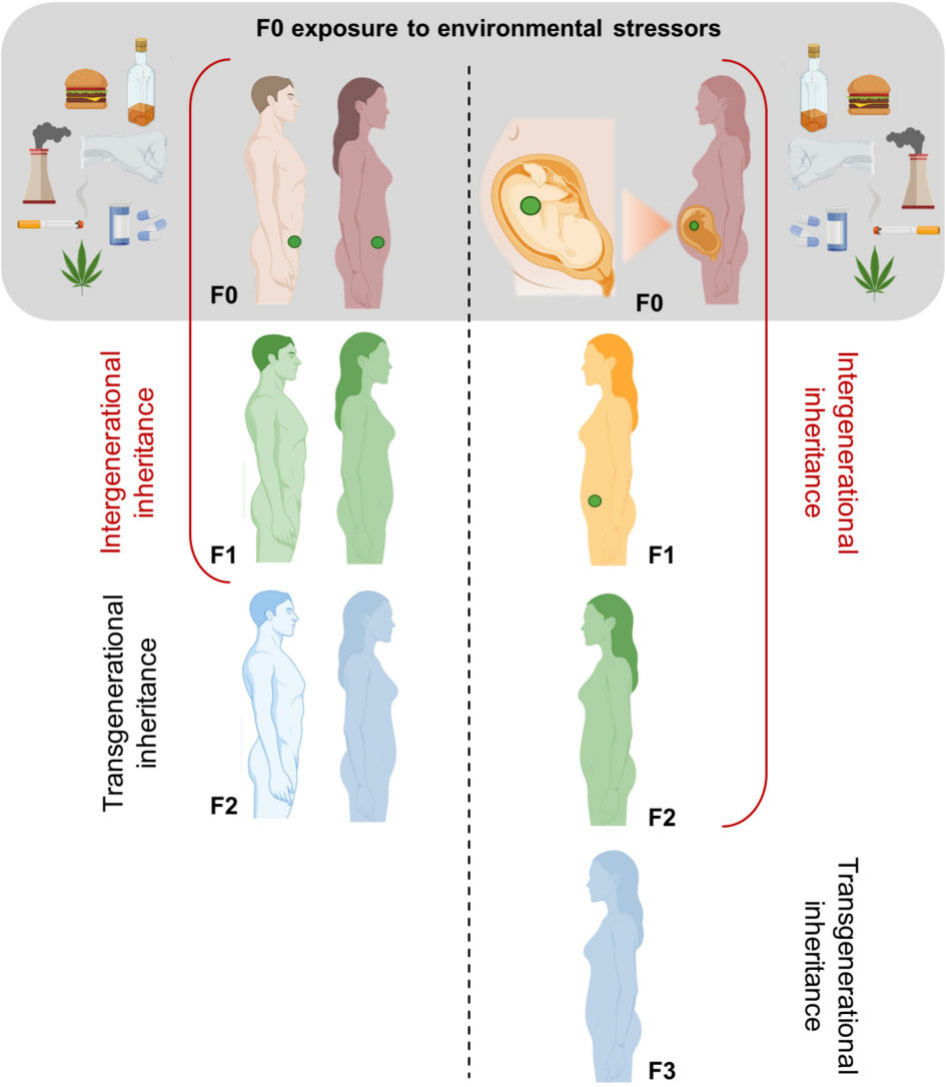
Intergenerational and transgenerational inheritance after exposure to environmental stressors. If a gestating mother (F0, right side) is directly exposed to environmental stressors, exposure may also affect the developing foetus (yellow) and its developing germ line (green), resulting in intergenerational effects (possible inheritance) in the F1 and F2 generations, since the affected generations were directly exposed. The third generation (F3, blue) is the first nondirectly exposed generation; therefore, an observed phenotype in F3 (or beyond) could result from transgenerational epigenetic inheritance. If a male or nonpregnant female (F0, left side) is directly exposed to environmental stressors, germ cells may also be directly affected, influencing the F1 generation (green). The F2 generation would be the first not directly exposed to trauma/stressors, and the appearance of the phenotype in F2 (or beyond) could result from transgenerational epigenetic inheritance. Nonetheless, to confirm transgenerational epigenetic inheritance, it is essential to exclude the effect of other confounding factors for a germline-mediated mechanism. Created using BioRender.com.

As shown in Fig. 2, a clear sex-specific distinction exists around intergenerational inheritance; in exposed males or nonpregnant females, it spans F0 to F1 and covers up to F2 in the case of exposed pregnant females (F0–F1–F2). Similarly, transgenerational inheritance started at F2 in exposed F0 males or nonpregnant females and at F3 in exposed F0 pregnant females.

Apart from the inter- or transgenerational transmission of the observed phenotype, there are sensitive phases of life during which traumatic stress and negative life experiences may be transmitted and impact an individual’s behaviour, cognition, and psychological/emotional development [1–4]. Their impact is greatest during the period from conception to age two, that is the first 1000 days of life. During this period, appropriate physical and psychological development of the foetus is implemented to allow the child to achieve correct growth and healthy survival [87].

During pregnancy, the foetus, or in early postnatal life, the infant, is characterized by intense neurogenesis, and inadequate stress-responsive pathways or aberrant neuroendocrine dynamics may affect balanced growth progression [88, 89]. Cognitive, behavioural, or physiological dysfunction may involve epigenetic mechanisms, such as DNA methylation, histone modifications, and noncoding RNAs (ncRNAs), which, although nongenetic, can be transmitted to subsequent generations [2]. This type of inheritance, called meiotic epigenetic inheritance (MEI), includes both intergenerational and transgenerational epigenetic inheritance [3, 26].

The transgenerational effects of traumatic events experienced in both early and adult life are observed in mammals, and the observable consequences may be summarized as behavioural abnormalities, mental disorders, or pathological dysfunctions, such as immune depression, autoimmune diseases, cancer, and cardiovascular and metabolic diseases [1–4, 6]. Remarkably, the limited number of human investigations has been circumvented by animal studies that support the transmission of acquired traits across generations. Accordingly, studies on behavioural and metabolic phenotype transmission showed observable phenotypes up to the fourth to fifth generation in a mouse model of paternal postnatal trauma, which were less evident in the matriline (i.e. up to the second generation), with attenuated symptoms in the sixth generation [90–92].

There are also observations of the existence of inter- and transgenerational sequences of behavioural adaptations in response to traumatic stressors and evidence of the transmission of adaptive actions expressed as physical or emotional flexibility aimed at enhancing or counteracting trauma responses [93]. Moreover, the effects of postnatal traumatic stress can be transmitted to the offspring independently of the germline, and in this case, it involves social transmission and the acquisition of behavioural or physiological patterns from parent(s) to offspring.

As recently hypothesized and suggested by the Genetic/Epigenetic Mother/Child Dyad Study (GEMCDS), offspring may epigenetically suffer from the maternal in utero environment during embryo/foetal development, and complex diseases such as leukaemia, blastoma, and neurodevelopmental disorders can also appear during childhood by interacting with their specific genetic backgrounds [94–96]. An extreme consequence of abnormal epigenetic maternal environments is pregnancy loss due to alterations in blastocyst implantation or embryonic development [97].

Understanding the emotional and psychological evolutionary bases of transgenerational epigenetic inheritance has strong potential clinical implications, and its impact on the psychological and emotional development of the offspring of female victims of violence is relevant.

Investigating the complex relationship between epigenetic mechanisms, stress, and affective disorders is mainly directed towards exploring how early life experiences and coping mechanisms may contribute to mood disorder susceptibility. Epigenetic factors are associated with maladaptive responses to stress and the occurrence, development, and progression of psychiatric and affective disorders [98]. Among several stress factors, female violence or trauma, work-related stress, and severe dietary deficiencies suggest that specific stress-related gene families are often epigenetically modified and contribute to specific affective disorder manifestations. Interestingly, whereas active coping is considered positive and correlates with better outcomes, emotion-focused coping may be responsible for unrestrained depression or manic episodes. Transgenerational epigenetic inheritance may have been selected during evolution to allow an individual to pass on experienced information received from the environment to offspring through an adaptive non-Mendelian inheritance mechanism [99].

Finally, considering the complexity of demonstrating direct epigenetic inheritance of trauma in humans and the limited available studies, to ascribe an epigenetic-mediated mechanism, there is a need for rigorous methodologies and systematic investigation of several generations, as reported in a recent publication on Syrian refugees and the intergenerational epigenetic violence signature [5, 47]. It is likely that genetic predispositions and environmental stimuli modulated by epigenetic mechanisms, together with individual stress responses, are the main causes of psychological and emotional development. Therefore, understanding these mutual interactions is essential for developing targeted interventions and personalized treatments for patients with post-traumatic mood disorders.

## Post-traumatic stress disorder

Traumatic stress relies on responses to physically, mentally, or emotionally disturbing events or abuse involving a threat to one’s physical or personal integrity. Trauma and stress responses may develop in an acute form or persist as a chronic condition for more than one month up to decades and are therefore classified as PTSD. Long-term traumatic stress can lead to mental, psychological, and emotional dysregulation; however, there is a growing concern about the impact of PTSD on physical health, longevity, and reproductive health.

From a biological perspective, although differences in individual responses and different sexes exist [100] and may be ascribed (but not limited) to genetic and epigenetic factors [101], PTSD seems to result from an abnormal and persistent response to stressors, leading to the concomitant alteration of key pathways, such as the endocrine and neuroendocrine systems [102]. Common symptomatic features refer to primary areas that can be schematized as intrusive symptoms (e.g. reminders, recurrent memories, and flashbacks), escape symptoms (e.g. amnesia, confusion, and dissociation), and activation symptoms (e.g. hyperarousal behaviour, anxiety, and anger) [101–104]. However, besides brain and psychological rebounds, several dysfunctional biological features characterizing patients with PTSD may increase the risk of developing physical comorbidities.

Among the biological pathways involved in regulating maternal–foetal interactions, the GABA and HPA axes play a leading role in representing major neuroendocrine systems, mediating the integrated homeostatic response to stressors by regulating several physiological processes [105]. We refer to a complex interrelated stress response system in which composite networks, including the sympathetic–adrenal–medullary (SAM) axis, hypothalamic–pituitary–gonadal (HPG) axis, endocrine and immune systems, and diffuse systemic systems, occur [106–108]. In summary, HPA-axis activation induces systemic changes, including immune responses, gastrointestinal activity modulation, energy balance, and regulation of autonomous activities, such as blood pressure and cardiac function [109]. In the presence of stressors, there is an increase in corticotrophin-releasing hormone (CRH) from the hypothalamus, which stimulates adrenocorticotropic hormone (ACTH) release, leading to the production of glucocorticoids (e.g. cortisol) by the adrenal glands. Importantly, reciprocal negative feedback loops exist between ACTH and cortisol production to ensure the fine regulation of their activities and rapid resolution of stress-induced HPA activation, along with other brain regions involved in the HPA axis. Once the stressor is resolved, the entire system returns to baseline conditions with the restoration of homeostasis and normal functioning of the cardiovascular, immunological, metabolic, reproductive, and digestive systems. Therefore, an acute HPA response to stressors is evolutionarily beneficial. In contrast, continuous stimulation of these integrated circuits by chronic traumatic stress results in dysregulation of the HPA axis, with associated pathological consequences.

Studies on patients with PTSD show an association with HPA system dysregulation, and long-term cortisol has been suggested as a potential biological marker of PTSD; temporal variations and individual differences may account for changes in the stress response [110]. Several studies on subjects with PTSD have reported low basal cortisol levels [111, 112], although HPA-axis dysregulation can also result in increased cortisol secretion, and transitions from hyper- to hypocortisolism may also be involved [111, 113]. Irrespective of the type of HPA-axis dysregulation, alterations in cortisol production are associated with PTSD, and hair cortisol concentration has been proposed as an index of cortisol levels integrated over the time of hair growth (i.e. several months), allowing ‘retrospective’ evaluations of the trauma impact and the role of pretrauma cortisol levels [114, 115]. However, the direct relationship between cortisol levels, neuroticism, and PTSD and the ‘direction’ of this association are still under debate [112, 116].

Besides HPA system dysregulation, other pathways may be involved in PTSD, including mineralocorticoid expression and function, which play a role in basal HPA-axis regulation, trauma management and re-elaboration, stress resilience, and vulnerability [117]. In addition, neuropeptides such as oxytocin and arginine vasopressin (argipressin) are mediators of emotional behaviour that may evolve into mood disorders, anxiety, and psychopathological conditions [118]. Dysregulation of the serotonergic and noradrenergic systems and responsivity to stress in subjects have been highlighted [119, 120] from a therapeutic perspective for trauma-related disorders [121].

Traumatic stress can differentially affect individuals based on (but not limited to) ethnicity, race, gender, and sex, modulating PTSD pathophysiology, prognosis, and other post-traumatic effects. It has been reported that rape, other sexual assault, and being stalked represent the most common traumas with a high risk of PTSD development (13.1%, 15.1%, and 9.8%, respectively), with the comprehensive type of ‘intimate partner sexual violence’ reported in nearly 42.7% of all PTSD subject-years [122]. Regarding sex and sex-related differences, PTSD is diagnosed more frequently in females than in males, and females maintain chronic symptoms of selected risk factors for PTSD severity longer than males, even after experiencing comparable traumas [123]. Acute stress disorder, neuroticism, lifetime sexual assault exposure, anxiety sensitivity, and pretrauma anxiety contribute to defining the link between sex and PTSD severity, suggesting the involvement of sex-related mechanisms [123]. Additional sex- and sex-specific factors potentially involved in PTSD onset/severity include specific sexualized experiences or biological conditions, such as hormonal fluctuations, pregnancy, and menopausal transition [124].

It has been highlighted that the endocrine/neuroendocrine dysfunction involved in PTSD is linked to chronic inflammation, characterized by alterations in the balance between inflammatory and anti-inflammatory cytokine profiles, altered immunity, changes in the gut microbiome, and disruption of the microbiota-gut-brain axis homeostasis [125, 126]. These underlying conditions result in an increased risk and prevalence of chronic diseases, including diabetes/cardiometabolic diseases [127, 128], cardiovascular events [129, 130], accelerated cognitive decline, depression, and an increased risk of death, reflecting distinct basal sex characteristics [131–135].

Misalignment of the appropriate temporal organization of temporal biological processes resulting from traumatic stress exposure may be an additional hallmark of PTSD. Circadian dysregulation affects key properties of the neuroendocrine, immune, and autonomic nervous systems, leading to stress-related disorders, increased stress sensitivity and vulnerability, and an increased risk of disease development [136, 137]. As a paradigm example, cortisol secretion is affected by several factors, including circadian rhythms, suggesting that there may be correlations between chronotype/circadian dysregulation and post-traumatic stress. Therefore, a better understanding of the mechanisms underlying circadian dysregulation and its role in PTSD could provide new insights into the mechanisms of PTSD [138]. Because current PTSD treatments are mainly psychotherapy and pharmacotherapy and no recognized PTSD biomarkers have been translated to the clinic to date [104], studies on innovative PTSD biomarkers are needed to better understand disease development and individual susceptibility, resilience, or coping mechanisms to establish preventive and therapeutic strategies in stress-exposed individuals [139].

## Medicolegal aspects of violence against women

Violence against women, including sexual violence and IPV, through feminicides, is a global phenomenon with significant public health implications. When current or former intimate partners commit it, the crime is classified as an IPV.

Gender violence, and in extreme cases, feminicide, continues to be a high-priority issue for the medical-legal and scientific communities to identify prevention programmes that can avoid the most serious events. As a paradigmatic example, female victims of violence end up in the emergency room more frequently, and the risk is that if violence is not promptly hypothesized and mistreatment is not suspected, appropriate measures will not be taken to protect the health and life of the victim. From a medicolegal perspective, it is essential to collect precise and detailed anamnesis and history of the victim so clinical forensic medicine can lead to the expression of a diagnostic judgement, differentiating between mistreatment and domestic violence [140]. Moreover, it is essential to inform victims that, based on the injuries they sustain and the details they provide, it is possible to predict whether the risk of future feminicide is low, medium, or high.

One of the most important problems in studying the feminicide phenomenon is the inhomogeneity of the data collected. Recent studies focusing on the identification and comparison of the medicolegal patterns of IPV in Europe show that no strong distinctions exist among countries and that certain IPV features are shared at the European level (data from Germany). One exception is the higher prevalence of firearm-related IPV in Italy [141]. A common characteristic shared worldwide is that the rates of IPV increased during the COVID-19 pandemic because of the lockdown [142].

The WHO defines feminicide as the intentional murder of a woman because of her sex or gender [143]. This description may be improved by a medicolegal definition of murder perpetrated because of a complete failure to recognize the victim’s rights [144]. The UNODC recently proposed a network cooperation aimed at facilitating the identification of feminicides among the total number of female homicides, regardless of age [145]. These data (i.e. a set of variables concerning both the victim and perpetrator and the context of the violence) can be further standardized using artificial intelligence and statistical approaches to produce relevant shared databases.

The available data, which are indicative though still preliminary, reveal that common features we observe are ‘an aggression aimed mainly at the face, as if one wants to erase the identity of the victim; at the mouth and the oral cavity, as if one intends to deprive her of word; to the neck, to symbolize the supremacy of the executioner over the fragility of the victim; and to the breast region and pubis, as a clear symbolic target of an attack on the victim’s sexuality’. Furthermore, there is the phenomenon of ‘overkilling’ in which the blows inflicted numerically exceed those sufficient to cause death [144, 146].

Predictably, the largest number of homicides involving women are committed by a current or former intimate partner [11]. Other types of feminicides are those defined as ‘honour-related feminicide’, being killed by a family member(s), being male or female, for an assumed sexual or behavioural transgression driven by ancestral wrong cultural heritage. Finally, violence committed by someone without an intimate relationship with a woman or girl should be considered [143].

Unfortunately, few European countries have available feminine databases (i.e. Italy, the UK, Spain, and Serbia); nevertheless, medicolegal comparisons are extremely useful for finding patterns and relevant correlations among countries to develop evidence-based policy strategies for prevention purposes [146–150]. Applying the same methodology to all investigated cases could be of great help in identifying which characteristic examined can be considered at greater risk of violence turning into a feminicide. Finally, the main goal is to create a common shared database including the epidemiological, clinical, and medicolegal characteristics of violence, including the biological and genomic features of criminals, to better and more quickly identify the culprit. Forensic pathology research can help develop targeted prevention strategies to protect women from this wave of violence.

An additional aspect to consider is the risk of a ‘structured’ sex-based inequality that may normalize and therefore exacerbate the dynamics of dominance and violence [151]. In Western countries, there is a general and diffuse stigma that limits the reporting of violence. The negative outcomes of these actions are emerging as targets for dedicated social and political interventions to lower barriers and facilitate access to sexual violence resource centres [152]. Finally, governments should prioritize the role of sexuality education and gender-inclusive protocols and services to contain and eradicate these phenomena.

## Discussion

Violence against women has ancient and historical origins. More than 2000 years ago, Roman law gave men the power of life and death over their wives. The ‘rule of thumb’, which allowed a man to punish his wife and children using a stick no wider than his thumb, was enforced in England and America until the late nineteenth century. This incorrect message has left deep scars on the psyche, mind, and physique of humans. Another type of ‘scars’ is that created in the genome of women victims of violence, a dramatic and actual concern that has scarcely been investigated from a biological and molecular perspective.

Many biological systems respond to environmental stressors by modifying their susceptibility to complex phenotypes and diseases. This biological cascade may include hormonal changes, oxidative stress, and epigenetic stress adaptation, as demonstrated in cancer, cardiovascular diseases, sensorineural hearing loss, and other complex phenotypes [153–156]. Among the several actions responsible for epigenetic modifications, exposure to violence has recently been considered an urgent primary phenomenon to be investigated, considering the hypothesized transgenerational effects of trauma [5, 47, 90].

**Figure 3.**
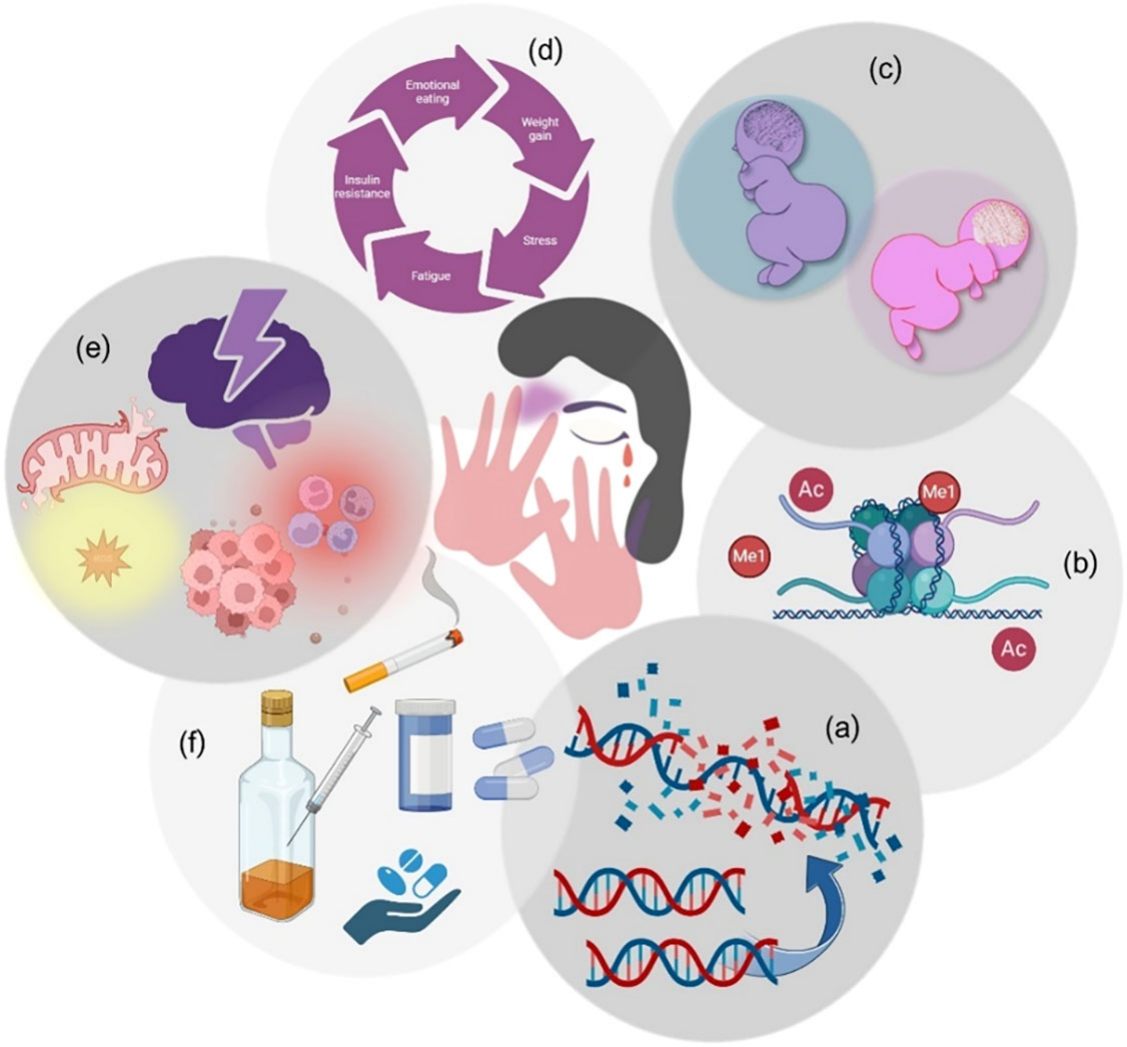
Snapshot of the vicious relationships among violence, epigenetics, and health. Violence against women may have direct and indirect effects on their psychological and physical status, well-being, and health. The resulting epigenetic modifications may (a) affect DNA structure and functionality, putting women’s health at risk; (b) alter methylation and acetylation of the relevant epigenetic mechanisms involved in traumatic stress; (c) generate transgenerational trauma effects, including traits of depression, anxiety, helplessness, and anger in newborns; (d) favour eating disorders and increase the risk of obesity and diabetes; (e) cause chronic stress response, HPA activation, high oxidative stress, and inflammation, increasing the risk of chronic diseases and death; and (f) favour the engagement of harmful behaviours. Created using BioRender.com.

The need to understand the epigenetic impact of violence and its essential mechanisms may improve future treatment strategies and stimulate personalized sex-specific care for patients and survivors [100, 135, 157]. This applies not only to visible physical wounds but also to hidden psychological signs that epigenetically mark the genomes of survivors. If not recognized early, due to the lack of biological or molecular informative biomarkers, this condition may lead to PTSD over the short, medium, and long term, manifesting as mental or physical distress, anxiety, depression, immunity, metabolism, cardiovascular diseases, and compromising the reproductive health of women by altering normal hormone homeostasis. Epigenetic imbalances may affect the quality of life of surviving women, causing higher morbidity and mortality and an increased risk of chronic diseases [158–161]. Moreover, additional indirect health problems may arise since survivors become more prone to unhealthy and harmful behaviours, such as substance and alcohol abuse (Fig. 3) [61, 162].

The existing literature, after approaching the phenomenon of violence against women from the perspective of epigenetic modifications and PTSD in survivors, demonstrated that specific epigenetic changes induced by major traumas or violence are comparable to those observed in war veterans or survivors of natural disasters or of maternal holocaust [49, 84, 163]. Similarly, the main results predominantly focused on the general concept of trauma epigenetics in child victims of violence. However, studies on adult women are limited, and the few published data have been recently revised and discussed in comprehensive reviews [61, 62]. These studies have a common epigenetic approach for investigating the mechanisms of global genome methylation, including the analysis of single-gene methylation, often of candidate gene promoters, or whole-epigenome analysis.

In this context, the most investigated genes are those related to PTSD, belonging to the GABA and HPA axis, cortisol metabolism, brain pathways, and plasticity, and are involved in neuroendocrine stress responses; dopamine, norepinephrine, and serotonin signalling; apoptosis; insulin secretion; reproduction; and foetal growth (e.g. *MAOA, BRSK2, ADCYAP1, BDNF, DRD2, IGF2, H19*). Moreover, additional investigated genes were those involved in other key functions, such as neuropeptide binding, immunoregulation, histone deacetylase/demethylase, inflammatory response, and serotonin uptake (e.g. *CRHR1, FKBP5, KDM1A, NR3C1, PRTFDC1, SLC6A4*), yielding interesting but preliminary or not completely replicated findings. Differences in the methylation of promoters or specific regions of these genes have been observed in female victims of violence. The association between epigenetic markers and exposure to violence differed among offspring depending on whether the violence occurred before or during pregnancy. This finding remarkably supports the hypothesis that the main common mechanism is direct exposure and that we are far from a definite recognition of demonstrating transgenerational inheritance; however, well- and rigorously designed studies can solve this intricate problem [5, 47].

A subtle but equally important issue to consider is the need to distinguish between sexual violence and IPV, since numerous studies have focused on indiscriminate sexual violence without making distinctions. However, the enrolment of women who are victims of violence in prospective studies is difficult because we not only need their informed consent and interviews but also biological samples at different times to evaluate any possible change or progression of potential novel epigenetic modifications. It should be considered that these women are shocked, traumatized, and vulnerable, and are at risk of a complete physical and psychological collapse. Therefore, medical professionals and paramedics must take immediate treatment actions before describing and proposing the study to victims and obtaining their consent to collect biological samples. This type of study and follow-up is challenging due to a plausible and comprehensible high dropout rate for these fragile and vulnerable women because it is difficult for them to return to the hospital and live through the traumatic experiences they have experienced in the past.

In the concept that nature and nurture both contribute to individual differences, the epigenome lies at the branch point between the genome and the environment, and it is susceptible and sensitive to external (exogenous) and internal (endogenous) stressors, being contextually considered a passive target or an active modulator of the specific mechanisms adopted in response to the same trauma [27]. Therefore, another aspect to consider during epigenetic investigations is the evaluation of possible concomitant or preceding situations that can confound or mask the results as the cause of epigenetic imbalance. Among these vulnerable individuals, there is a personal or familial situation of chronic stress, previous mistreatment or violence, a high prevalence of drug and alcohol habits, and other subtle behavioural disorders [160, 164–166]. Moreover, in real life, it is less frequent to find a healthy girl or woman experiencing violence or rape for the first time in the absence of concomitant interfering situations, who may therefore exhibit epigenetic modifications exclusively ascribable to that specific violent event. These actions should be considered separately and analysed epigenetically to avoid additional confounding factors that could alter observed findings. Moreover, the lack of appropriate controls for use as a reference, such as biological samples or medical/psychological history collected before the violence, makes epigenetic comparisons and accurate analyses difficult to discriminate between epigenetic marks written in the victim’s DNA before or after violence. Finally, interaction between epigenetic markers (e.g. DNA methylation *versus* telomere attrition) should be considered as informative predictors of individual basal frailty, coping, and resilience [167, 168].

Accordingly, a wide range of epigenetic responses has been observed in PTSD victims. This depends not only on the specific physical or psychological trauma experienced but also on the personal epigenetic landscape of each individual. The genome and epigenome mutually interact, and crosstalk between their components determines and influences global health outcomes. Similarly, the degree of generational transmission depends on whether the PGCs epigenome is marked by environmental stressors, such that after meiosis, they can transmit and mitotically maintain the modifications up to successive mature gametes or embryos. This point is crucial to avoid incorrect designation of the inheritance of phenotypes despite the mere persistence of the trait because of a direct exposure effect. Finally, future epigenetic interventions aimed at having a curable (epi)genome will raise dilemmas and fear within society, and dreading them that this will change people’s identities. The public needs to be well educated about these new technologies to avoid situations where new technologies are hindered, as was the case when gene therapy was first introduced for genetic disease treatment or for the more recent anti-SARS-Cov-2 mRNA-based vaccines, which were a source of alarm among clinicians, researchers, politicians, and the population [169–171]. In addition, although epigenomic actions theoretically offer ethical advantages because they are generally considered nonheritable, there are concerns regarding their potential transgenerational effects in humans. Thus, ethical and regulatory evaluations are crucial, considering the recent understanding of transgenerational epigenetics in mammals and humans [28, 29, 171].

A multidisciplinary tailored healthcare approach, including but not limited to geneticists and psychiatrists, by the identification and modification of specific epigenetic marks in survivors could counteract the underlying mechanisms responsible for PTSD establishment. This may avoid its appearance and long-lasting effects, reducing the chance of transmitting complex disorder risk factors to the offspring. The fascinating perspective that epigenetic marks can be reversed by appropriate social, psychological, and pharmacological interventions and treatments provides crucial insights into ‘the curable epigenome’, i.e. how to erase the trauma of violence and re-establish physical and mental well-being in women survivors and other victims of violence.

## Established facts, emerging hypotheses, and future actions

Traumatic experiences activate a complex series of molecular mechanisms that help cope with stress. This translates into individual bioadaptability to adversity and different health consequences, emphasizing the dynamism of adaptation to trauma and the great opportunity for translational research to develop effective intervention strategies. Epigenetic variability is associated with different environmental adversities, allowing for distinct resilience and adaptability. Epigenetics, including age-, sex-, environment-, drug-, and lifestyle-dependent DNA methylation, are key factors in understanding trauma responses and promoting well-being by integrating psychological and biological adaptations. A deeper understanding of these molecular processes offers the opportunity to build biologically adaptive frameworks and shift public health policies from restorative to long-term resilience. Physical, psychological, and biological trauma must be addressed through innovative interventions for vulnerable populations, particularly women, elderly, children, and adolescents. Understanding the epigenetic changes may provide a biopsychosocial perspective for future culturally sensitive interventions and evidence-based actions aimed at promoting resilience in novel epigenetic settings [27].

The transgenerational inheritance of epigenetic markers caused by environmental stressors may influence the health outcomes of multiple generations. Continuous exposure to daily environmental stressors has led to the evolution of humans. From the search for food and water, to escaping wild animals, to conflicts between tribes and wars, millennia have shaped and ‘scarred’ the epigenome. In addition, famines, such as the Dutch famine in the Western Netherlands during the winter of 1944–1945 that affected the epigenome by accelerating the biological clock for decades after prenatal stress exposure [172], pandemics, or natural events have epigenetically marked the genome as fundamental to human evolution.

Of note, although established in plants and some animals, epigenetic inheritance in mammals, especially humans, remains debated, with several emerging hypotheses and ongoing controversies. The physiological epigenetic mechanisms involved in normal development and gene regulation (DNA methylation, histone modifications, and ncRNAs) are the same factors used by the environment to induce novel epigenetic changes and marks. Accordingly, changes that can be passed down to offspring and potentially affect development and health have been demonstrated in various organisms, such as invertebrate models (e.g. *C. elegans* and *Drosophila*), in which inherited epigenetic changes across multiple generations have been shown and can take several generations to be fully re-established [173–175].

Emerging hypotheses and ongoing research refer to the role of germline reprogramming, which also involves mature gametes. As previously reported, mammalian germ cells undergo extensive reprogramming during development to erase most epigenetic marks; however, some cells and genomic regions may evade this process by the so-called ‘escapee’ genes and loci that apparently resist DNA demethylation in PGCs [176]. This may partially account for transgenerational transmission, whereas intergenerational mechanisms are because of direct exposures. Researchers are investigating the mechanisms of epigenetic inheritance, including the transmission of epigenetic information through sperm and eggs, and roles of specific ncRNA and histone retention [177].

From a novel perspective, transgenerational epigenetic inheritance may offer a mechanism for organisms to adapt to changing environment, although the potential for both beneficial and detrimental effects remains to be explored. There are several controversies and challenges in determining the extent of epigenetic inheritance in mammals. A key point is to clarify the degree to which epigenetic information is transmitted across generations, especially in humans, and the lastingness of epigenetic changes, opening the way for the ‘curable epigenome’ concept. In human studies, this can be challenging because it is difficult to separate the effects of inherited genes from those of (inherited) epigenetic changes. Understanding transgenerational inheritance in humans also raises ethical questions about the potential risks and benefits for future generations, especially in the context of environmental exposure [178].

In conclusion, although the concept of transgenerational epigenetic inheritance is well established in some organisms, research on humans is ongoing, with both established facts and emerging hypotheses. This fascinating field actively explores the underlying mechanisms, extent, and implications for health, helping us understand heredity and evolution.

## Purpose and methodology

This narrative review aims to summarize the potential connections between biological epigenetic mechanisms and physical or psychological violence in women by evaluating current research/evidence, providing context and insights, and identifying knowledge gaps. The relevance of transgenerational epigenetic inheritance and its possible clinical implications are also discussed. This work inspires new research from scientists with different levels of expertise as ‘agents of change’ in this developing and pressing issue that affects the health and well-being of women and their children worldwide. The present review was based on a comprehensive literature search of the PubMed and Scopus online databases conducted between November 2024 and June 2025 using a custom-made search term list to guarantee an inclusive selection of relevant studies on the topic. However, the proposed selection method has certain potential deficiencies. Studies were selected based on their relevance to the key subjects explored in the review, including (1) genes and epigenetic markers of violence, (2) epigenetics and transgenerational impact of violence and trauma, (3) violence and biology of PTSD, and (4) medicolegal aspects of violence against women. Custom-made search terms list by key areas included ‘genes and epigenetics of violence’, ‘intimate partner violence’, ‘women violence’, ‘gender-based violence’, ‘transgenerational impact of violence’, and ‘post-traumatic stress disorders’.

A large portion of the international literature focuses on epigenetic modifications caused by violence against children or adolescents, neglecting adults, including women. The main approaches addressed the genome methylation of genes involved in the stress response and associated PTSDs. Several limitations and inhomogeneity are present in the published studies, mainly accounting for the biological sample considered (e.g. peripheral blood *versus* buccal swabs), the wide age range of females enrolled (e.g. adult *versus* childhood), the time elapsed between violence occurrence and collection of biological samples, the type of violence (e.g. sexual assault and/or physical and/or psychological violence), and cases of women who had experienced violence during infancy [61–64]. Finally, the small number of participants and high dropout rate were significant limitations of these studies.

Considering the abovementioned limitations and concerns, future studies should plan more structured methodological approaches to address crucial issues such as the timing of biological sample collection, structured clinical anamnesis, personal/familiar history of subjects, longer follow-up to evaluate any epigenetic modification, and analysis of multiple generations when possible.
